# Mixed-type gastric carcinomas exhibit more aggressive features and indicate the histogenesis of carcinomas

**DOI:** 10.1007/s00428-007-0572-7

**Published:** 2008-02-12

**Authors:** Hua-chuan Zheng, Xiao-han Li, Takuo Hara, Shinji Masuda, Xiang-hong Yang, Yi-fu Guan, Yasuo Takano

**Affiliations:** 1 Department of Biochemistry and Molecular Biology, College of Basic Medicine, China Medical University, Shenyang, China; 2 Department of Diagnostic Pathology, Graduate School of Medicine and Pharmaceutical Science, University of Toyama, Toyama, Japan; 3 Kouseiren Takaoka Hospital, Takaoka, Japan; 4 Division of Pathology, Shengjing Hospital of China Medical University, Shenyang, China

**Keywords:** Gastric carcinoma, Mixed type, Lauren's classification, Pathobiological behaviors, Carcinogenesis

## Abstract

To investigate the pathobiological behaviors of gastric mixed-type (MT) carcinomas and gastric carcinogenesis, the clinicopathological characteristics of MT carcinomas were analyzed and compared with intestinal-type (IT) and diffuse-type (DT) carcinomas. The expression of Ki-67, caspase-3, p53, fragile histine triad (FHIT), maspin, extracellular matrix metalloproteinase inducer (EMMPRIN), vascular growth factor (VEGF), MUC-2, 4, 5AC and 6, CD44, E-cadherin, β-catenin, and phosphorylated glycogen synthase kinase 3β-ser^9^ (P-GSK3β-ser^9^) was examined on tissue microarrays using immunohistochemistry. It was found that MT carcinomas exhibited large size, deep invasion, frequent local invasion, and lymph node metastasis in comparison with IT and DT carcinomas (*p* < 0.05). All the markers except MUC-5AC showed higher expression in IT than DT carcinomas (*p* < 0.05). The expression of maspin, EMMPRIN, VEGF, MUC-4, and membrane E-cadherin was stronger in MT intestinal than diffuse component (*p* < 0.05). Immunoreactivities to Ki-67, EMMPRIN, and VEGF were weaker in IT carcinoma than in the MT intestinal portion (*p* < 0.05), while the opposite was true for CD44, MUC-2, and MUC-6 (*p *< 0.05). The MT diffuse component displayed a higher expression of FHIT, VEGF, and P-GSK3β-ser^9^ than DT carcinoma (*p *< 0.05). The accumulative survival rate of the IT carcinoma patients was higher than the other types (*p *< 0.05). The invasive depth, venous invasion, lymph node, peritoneal or liver metastasis, and Lauren's classification were independent prognostic factors for gastric carcinomas (*p* < 0.05). These findings suggested that MT carcinomas were also indicated to be more aggressive than IT and DT carcinomas. Significant differences were observed in the proliferation, apoptosis, angiogenesis, mucin secretion, and cell adhesion between IT and DT carcinomas, whereas only a few of these characteristics showed differences between the MT intestinal and diffuse parts, thus suggesting that both the MT components might originate from the stem cells with similar genetic traits, but follow different histogenic pathways.

## Introduction

Gastric carcinoma is the second leading cause of cancer-related death behind lung carcinoma despite a worldwide decline in both its incidence and mortality since the late half of the twentieth century [11]. It continues to be a major health problem because of the slow decrease in incidence in Asia and high mortality of diagnosed gastric carcinoma in the West [23]. Generally, the prognosis of patients with gastric carcinoma is somewhat dependent on its histological type, such as intestinal type (IT) or diffuse type (DT) as defined by Lauren [16].

Although gastric carcinoma is a malignant tumor originating from the same gastric epithelium, its morphological features vary substantially among individual patients. Histologically, IT carcinoma principally includes papillary, well-differentiated, and moderately-differentiated or mucinous adenocarcinoma without signet ring carcinoma (SRC) cells, whereas DT mainly consists of the SRC, poorly differentiated and undifferentiated adenocarcinoma of WHO classification [7, 23]. Although Kushima and Hattori [12] firstly proposed an entity of gastric-type carcinoma and terminologically seemed homologous to the IT one, the carcinoma cells of this type closely resemble normal foveolar cells on the basis of tissue morphology and mucin properties and are considered to have been derived from foveolar hyperplasia or pyloric gland adenoma [12, 13]. Recently, our group also focused on Lauren's classification and found that IT carcinoma frequently occurred in old men, while DT was more frequent in comparatively young women. The latter group was more inclined to invasion into the muscularis propria, lymphatic invasion, and lymph node metastasis, and belonged to a higher TNM staging, in comparison to its IT counterparts. Further analysis demonstrated that IT gastric carcinomas with a more favorable prognosis were prone to high levels of proliferation and apoptosis and also always accompanied by a strong expression of the fragile histine triad (FHIT), phosphatase and tensin homology deleted from human chromosome 10 (PTEN), p53, and extracellular matrix metalloproteinase inducer (EMMPRIN) [30]. Additionally, many genetic and epigenetic changes, such as mutation, deletion, loss of heterozygosity, methylation, and microsatellite instability were differentially observed in both histological types of gastric carcinoma [27, 36]. These data indicate that different carcinogenic pathways exist for gastric IT and DT carcinomas. Although carcinogenesis is a multistage process which consists of a multi-factorial etiology resulting from gene–environment interaction, the intestinal type is frequently related to environmental factors like diet and predominates in areas with a high incidence of this disease. In contrast, the diffuse type is thought to be of genetic origin and evenly distributed worldwide [30, 24]. It is commonly believed that Lauren's classification is valuable for both the epidemiological studies and gastric carcinogenesis.

However, a small number of gastric carcinomas remain characterized as unclassified or mixed-type (MT) ones, which are comprised histologically of non-homogenous mixtures of IT and DT carcinomas. Stelzner and Emmrich [26] found that 28 MT carcinomas showed a deep infiltration of the gastric wall, frequent regional lymph node metastasis, and high staging in comparison to the other two types. Machado et al. [21] reported that inactivating E-cadherin mutations were exclusively observed in the diffuse component of the tumors, thus suggesting that MT carcinomas displayed phenotypic divergence. A recent study showed that bone-marrow-derived cells (BMDCs) could progress through metaplasia and dysplasia to intraepithelial cancer under the induction of the carcinogen, *Helicobacter pylori* [9]. Therefore, the analysis of distinct MT carcinoma components can be of remarkable pathogenic significance if it would be true that carcinoma cells originate from BMDCs. In the present study, 814 cases of gastric carcinomas were collected to further clarify the pathological characteristics of MT carcinoma and the gastric carcinogenesis pathways using a combination of tissue microarray (TMA) and immunohistochemical techniques.

## Materials and methods

### Subjects

A total of 814 gastric carcinomas were collected from surgical resection in Kouseiren Takaoka Hospital between 1998 and 2006. The patients with carcinomas included 573 men and 241 women (29–91 years, mean = 65.7 years). Among them, 312 cases were demonstrated with lymph node metastasis and 24 with liver metastasis. None of these cases underwent either chemotherapy or radiotherapy before surgery. All patients gave their informed consent for the use of tumor tissue specimens for clinical research and the University Ethical Committee approved the research protocol. All patients were followed up by consulting their case documents and through telephone interviews.

### Pathology

All tissue specimens were fixed in 4% neutralized formaldehyde, embedded in paraffin, and cut into 4-μm sections. These sections were stained by hematoxylin and eosin (HE) to confirm their histological diagnosis and other microscopic characteristics. The staging for each gastric carcinoma was evaluated according to the TNM system of the Internationale Contre le Cancer (UICC) indicating the extent of tumor spread [25]. The histomorphological architecture of the tumors was expressed according to Lauren's classification [7, 16, 30]. The growth patterns were divided into five groups based on a modification of Emmrich's method [26]. Briefly, group I showed a combination of two components with diffuse distribution. Group II displayed both components with the border clearly visible. Group III represented some signet cells in the intestinal and diffuse components. Group IV were mucinous carcinomas with signet cells. Group V exhibited separately intestinal and diffuse components in the gastric wall. In addition, the depth of invasion, lymphatic and venous invasion, and peritoneal dissemination were all determined.

### Tissue microarray

**Fig. 1:**
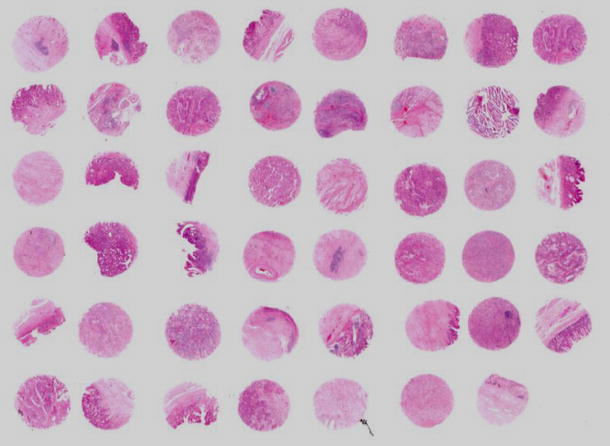
HE staining of TMA of gastric carcinomas

IT and DT carcinomas, as well as both components of the mixed type, were identified in HE stained sections of the selected tumor cases and a 2-mm-diameter tissue core of each donor block was punched out and transferred to a recipient block with a maximum of 48 cores using a Tissue Microarrayer (AZUMAYA KIN-1, Tokyo, Japan). Four-micrometer-thick sections were cut from the recipient block and transferred to poly-lysine-coated glass slides. HE staining was performed on TMA for confirmation of the tumor tissue (Fig. 1).

### Immunohistochemistry

Consecutive sections were deparaffinized with xylene, dehydrated with alcohol, and subjected to antigen retrieval by irradiating in target retrieval solution (TRS, DAKO, Carpinteria, UAS) for 15 min in a microwave oven (Oriental rotor Lmt. Co. Tokyo, Japan). Five percent bovine serum albumin (BSA) was then applied for 15 min to prevent non-specific binding. The sections were incubated with primary antibodies for 15 min, then treated with the anti-mouse or anti-rabbit Envison-PO (DAKO, USA) antibodies for 15 min. All incubations were performed in the microwave oven for intermittent irradiation as described previously [15]. After each treatment, the slides were washed with TBST (10 mM Tris–HCl, 150 mM NaCl, 0.1% Tween 20) three times for 1 min. The primary antibodies are summarized in Table 1. All immunostaining was visualized with 3, 3′-diaminobenzidine (DAB) with 5-min reaction and counterstained with Mayer's hematoxylin. Omission of the primary antibody was used as a negative control.

**Table 1:** Table 1 Primary antibodies used in this study

Names	Source	Company	Dilution
Ki-67	Rabbit	DAKO, Carpinteria, USA	1:25
Caspase-3	Rabbit	DAKO, USA	1:150
p53	Mouse	DAKO, USA	1:100
FHIT	Rabbit	Neomarkers, Fremont, USA	1:200
Maspin	Mouse	Novocastra, Newcastle upon Tyne, UK	Read-to-use
EMMPRIN	Mouse	Novocastra, UK	1:100
VEGF	Rabbit	Labvision, Fremont, USA	1:50
P-GSK3β-ser^9^	Rabbit	SAT, USA	1:300
MUC-2	Mouse	Novocastra, UK	1:100
MUC-4	Mouse	Novocastra, UK	1:100
MUC-5AC	Mouse	Novocastra, UK	1:100
MUC-6	Mouse	Novocastra, UK	1:100
CD44	Mouse	DAKO, USA	1:50
E-cadherin	Mouse	Takara, Otsu, Japan	1:100
β-Catenin	Mouse	Calbiochem, CA, USA	1:200

**Fig. 2:**
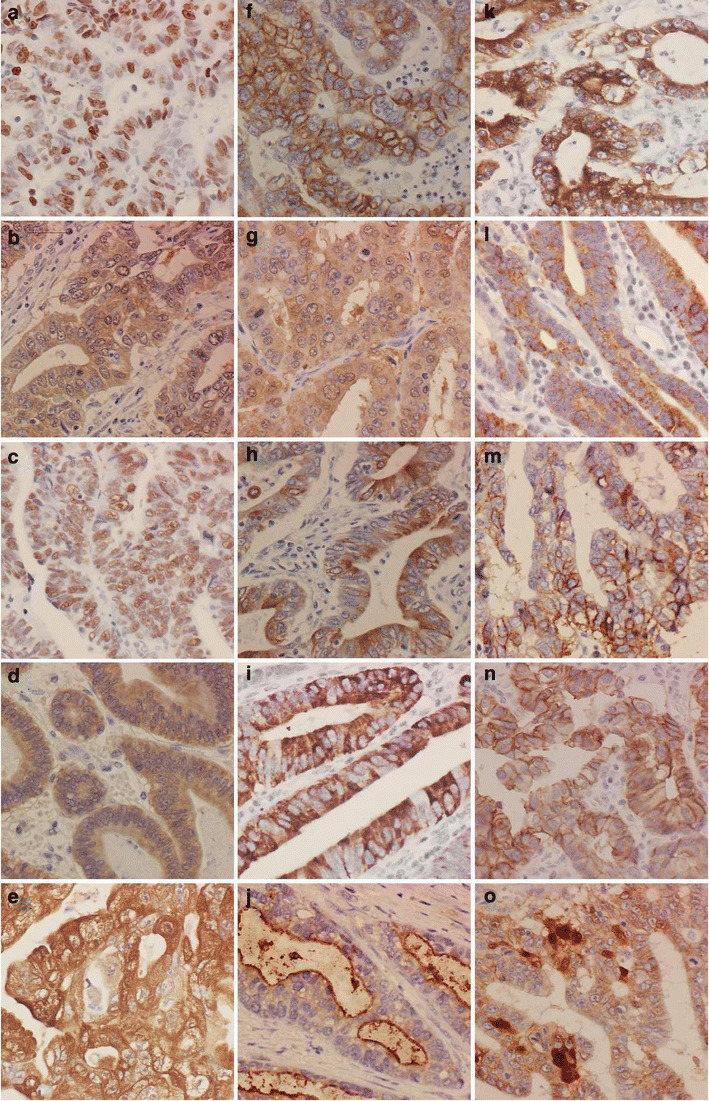
Immunostaining of TMA of gastric carcinomas. The immunoreactivity to Ki-67 (**a**) and p53 (**c**) was localized in the nucleus; FHIT (**d**), caspase-3 (**b**), VEGF (**g**), maspin (**e**), P-GSK3β-ser^9^ (**h**), MUC-2 (**i**), MUC-5AC (**k**), MUC-6 (**l**) were seen in the cytoplasm, EMMPRIN (**f**) and MUC-4 (**j**) were seen in the cytoplasm and the plasma membrane, CD44 (**m**), and E-cadherin (**n**) in the plasma membrane; and β-catenin (**o**) in the nucleus, cytoplasm, and plasma membrane. These molecules were strongly expressed in the intestinal-type gastric carcinomas

The immunoreactivity to Ki-67 and p53 was localized in the nucleus; FHIT, caspase-3, vascular growth factor (VEGF), maspin, phosphorylated glycogen synthase kinase 3β-ser^9^ (P-GSK3β-ser^9^), MUC-2, MUC-5AC, and MUC-6 in the cytoplasm; EMMPRIN and MUC-4 in the cytoplasm and membrane; CD44 and E-cadherin in the membrane; and β-catenin in the nucleus, cytoplasm, and membrane (Fig. 2). All evaluations were performed blindly by two independent observers (Takano Y and Zheng HC).

### Statistical analysis

The statistical evaluation was performed using Fisher's exact possibility to differentiate the rates and the Mann–Whitney *U* test to differentiate the means of the different groups. Kaplan–Meier survival plots were generated and comparisons between the survival curves were made with the log-rank statistic. The Cox proportional hazards model was employed for multivariate analysis. *p* < 0.05 was considered to represent a statistically significant difference. The SPSS 10.0 software program was employed to analyze all data.

## Results

### Clinicopathological characteristics of gastric IT, DT, and MT carcinomas

As summarized in Table 2, 415 IT cases were identified (51.0%), 221 DT (27.1%), and 178 MT (21.9%) among the 814 gastric carcinomas in this study. Patients with IT carcinoma were found to be significantly older than those with DT (*p* < 0.05). The male and female ratios in the carcinoma cases gradually decreased from IT to DT through the MT groups (*p* < 0.05). It was the same for lower TNM staging in the three groups (*p* < 0.05). The positive rate of peritoneal spread was higher in the DT and MT cases than IT (*p* < 0.05). MT carcinomas showed aggressive characteristics, such as large size, deep invasion, frequent local invasion, and lymph node metastasis, compared to the IT and DT (*p* < 0.05). There was no significance between the three types of gastric carcinomas in the incidence of the liver metastasis (*p* > 0.05).

**Table 2:** Table 2 Comparison of the clinicopathological features in gastric IT, DT, and MT carcinomas

Clinicopathological features	Intestinal-type carcinoma	Diffuse-type carcinoma	Mixed-type carcinoma
Case number	415 (51.0%)	221 (27.1%)	178 (21.9%)
Age (mean ± SD, years)	67.09 ± 10.73	62.03 ± 11.3*	65.80 ± 13.28
Sex (male:female)	324:91	125:96	124:54**
Tumor size (mean ± SD, cm)	3.82 ± 3.21	5.50 ± 6.95	5.56 ± 3.08**
Invasion into muscularis propria	153 (36.9%)	121 (54.8%)	123 (69.1%)**
Lymphatic invasion (+)	117 (28.2%)	83 (37.6%)	99 (55.6%)**
Venous invasion (+)	49 (11.8%)	32 (14.5%)	39 (21.9%)**
Lymph node metastasis (+)	113 (27.2%)	94 (42.5%)	105 (59.0%)**
Liver metastasis (+)	10 (2.4%)	8 (3.6%)	6 (3.4%)
Peritoneal spread (+)	14 (3.4%)***	22 (10.0%)	21 (11.8%)
TNM staging (O, I)	302 (72.8%)	69 (31.2%)	112 (63.0%)**

*p < 0.05 compared with intestinal- and mixed-type carcinomas

**p < 0.05 compared with intestinal- and diffuse-type carcinomas with both analyzed

***p < 0.05 compared with diffuse- and mixed-type carcinomas

### Pathological behaviors of gastric MT carcinomas with different growth patterns or histological distribution of components

According to the growth pattern, the intestinal and diffuse components diffusely distributed without absolute border in group I of the MT carcinomas, which occurred more frequently (60.0%, 106/178) than the other four groups. As indicated in Table 3, most groups III and V gastric carcinomas were generally of early stage with less local invasion, infrequent lymph node metastasis and low TNM staging. In this study, only four advanced gastric carcinomas belonged to group IV, among which three cases were women and all were accompanied with lymphangiogenic invasion, lymph node metastasis, and high TNM staging. Although the main histological component was different from that in lymph node metastasis, the statistical data revealed that there was histological consistency between primary and lymph node metastasis foci (*p* < 0.05). There were no remarkable differences in the pathological behaviors of MT carcinomas with different ratios of intestinal and diffuse portions (*p* > 0.05).

**Table 3:** Table 3 Pathological behaviors of gastric MT carcinomas

Clinicopathological features	*n*	Sex (male)	Invasion into MP	Lymphatic invasion	Venous invasion	Lymph node metastasis				Staging
						*n*	I>D	I=D	I<D	(O, I)
Growth pattern										
I	106	73 (68.9%)	82 (77.4%)	64 (60.4%)	26 (24.5%)	69 (65.1%)	32	14	23	35 (33.0%)
II	25	18 (78.3%)	21 (84.0%)	16 (64.0%)	7 (28.0%)	16 (64.0%)	5	5	6	7 (28.0%)
III	29	22 (75.9%)	11 (37.9%)	9 (31.0%)	3 (10.3%)	10 (34.5%)	4	3	3	18 (62.1%)
IV	4	1 (25.0%)	4 (100.0%)	4 (100.0%)	1 (25.0%)	4 (100.0%)	0	2	2	0 (0.0%)
V	14	10 (71.4%)	5 (35.7%)	6 (42.9%)	2 (14.3%)	6 (42.9%)	2	0	4	9 (64.3%)
Histological appearance										
Intestinal>diffuse	80	59 (73.8%)	54 (67.5%)	43 (53.8%)	20 (25.0%)	48 (60.0%)	30	8	10	33 (41.3%)
Intestinal=diffuse	20	15 (75.0%)	12 (60.0%)	9 (45.0%)	5 (25.0%)	10 (50.0%)	3	5	2	8 (40.0%)
Intestinal<diffuse	78	50 (64.1%)	57 (73.1%)	47 (60.3%)	14 (18.0%)	47 (60.3%)	10	11	26	28 (35.9%)

*MP* Muscularis propria; in lymph node metastatic foci: *I>D* intestinal>diffuse, *I<D* intestinal<diffuse, *I=D* intestinal=diffuse

### Immunohistochemical analysis of intestinal or diffuse components from three types of gastric carcinomas

Table 4 demonstrates that all the markers except MUC-5AC were expressed at higher levels in IT carcinomas than in MT ones (*p* < 0.05). The expression of maspin, EMMPRIN, VEGF, MUC-4, and E-cadherin was stronger in the intestinal component of MT carcinomas than in their diffuse counterpart (*p* < 0.05). Immunoreactivities to Ki-67, EMMPRIN, and VEGF were weaker in the intestinal-type carcinomas than in the intestinal component of the MT (*p* < 0.05), while it was the opposite for CD44, MUC-2, and MUC-6 (*p* < 0.05). In contrast, the diffuse components of the MT carcinomas showed greater expression of Ki-67, FHIT, VEGF, and P-GSK3β-ser^9^ than DT carcinomas (*p* < 0.05).

**Table 4:** Table 4 Immunohistochemical analysis in gastric IT, DT, and MT carcinomas

Biological markers	Intestinal-type carcinoma	Mixed-type carcinoma		Diffuse-type carcinoma
		Intestinal part	Diffuse part	
Ki-67	88/146 (60.3%)*^,^**	81/110 (73.6%)	78/110 (70.9%)*	57/119 (47.9%)
Caspase-3	84/152 (55.3%)*	52/119 (43.7%)	37/119 (31.1%)	24/115 (20.9%)
p53	76/151 (50.3%)*	37/118 (31.4%)	30/118 (25.4%)	25/120 (20.8%)
FHIT	90/150 (60.0%)*	63/113 (55.8%)	48/113 (42.5%)*	27/116 (23.3%)
Maspin	71/150 (47.3%)*	55/112 (49.1%)***	34/112 (30.4%)	38/120 (31.7%)
EMMPRIN	87/147 (59.2%)*^,^**	77/112 (68.8%)***	43/112 (38.4%)	32/117 (26.5%)
VEGF	95/151 (62.9%)*^,^**	90/114 (78.9%)***	61/114 (53.5%)*	31/121 (25.6%)
P-GSK3β-ser^9^	88/144 (61.1%)*	74/113 (65.5%)	67/113 (59.3%)*	48/116 (41.4%)
MUC-2	55/145 (37.9%)*^,^**	31/118 (26.3%)	27/118 (22.9%)	23/117 (19.7%)
MUC-4	58/153 (37.9%)*	39/118 (33.1%)***	21/118 (17.8%)	18/118 (15.3%)
MUC-5AC	84/148 (56.8%)	57/106 (53.8%)	50/106 (47.2%)	54/113 (47.8%)
MUC-6	60/146 (41.1%)*^,^**	26/117 (22.2%)	18/117 (15.4%)	9/123 (7.3%)
CD44	64/152 (42.1%)*^,^**	32/114 (28.1%)	31/114 (27.2%)	32/120 (26.7%)
E-cadherin	91/142 (64.1%)*	58/112 (51.8%)***	38/112 (33.9%)	43/119 (36.1%)
Membrane β-catenin	57/144 (39.6%)*	36/117 (30.8%)	26/117 (22.2%)	26/118 (22.0%)

*p < 0.05 compared with the diffuse-type carcinomas

**p < 0.05 compared with intestinal-part carcinomas

***p < 0.05 compared with diffuse-part carcinomas

### Patients' outcome with different gastric carcinomas

**Fig. 3:**
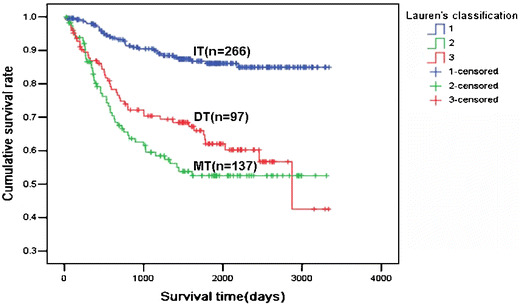
Relationship between prognosis of carcinoma patients' and Lauren's classification Kaplan–Meier curves of cumulative survival rate for the patients with gastric carcinoma according to Lauren's classification. *IT* Intestinal type, *DT* diffuse type, *MT* mixed type

Follow-up information for 500 carcinoma patients was used before 2002 for a period ranging from 5 days to 9.15 years (mean = 49.4 months). Figure 3 shows the survival curves stratified according to Lauren's classification. Kaplan–Meier analysis indicated that the patients with IT carcinoma had a higher cumulative survival rate than those with the DT and MT lesions (*p* < 0.05). Although the MT carcinoma patients' survival rate was comparatively lower than the DT ones, there was no statistical significance (*p* > 0.05). Multivariate analysis demonstrated that invasive depth, venous invasion, lymph node, peritoneal or liver metastasis, and Lauren's classification were independent factors for the poor prognosis of gastric carcinoma patients (*p* < 0.05; Table 5).

**Table 5:** Table 5 Multivariate analysis of the clinicopathological variables of gastric carcinomas

Clinicopathological parameters	Relative risk (95% CI)	*p* Value
Age (≥65 years)	1.224 (0.849–1.764)	>0.05
Sex (male:female)	1.255 (0.827–1.904)	>0.05
Tumor size (≥4 cm)	1.466 (0.862–2.493)	>0.05
Invasive depth (Ti_s,1_/*T*_2,3_)	2.945 (1.533–5.657)	<0.05
Lymphatic invasion (−/+)	1.394 (0.865–2.247)	>0.05
Venous invasion (−/+)	1.692 (1.097–2.608)	<0.05
Lymph node metastasis (−/+)	1.983 (1.111–3.537)	<0.05
Peritoneal spread (−/+)	3.197 (2.031–5.031)	<0.05
Liver metastasis (−/+)	5.248 (2.575–10.697)	<0.05
Lauren's classification (intestinal/diffuse/mixed)	1.351 (1.084–1.683)	<0.05

*CI* Confidence interval

## Discussion

Histologically, Lauren's classification of gastric carcinomas is based on the morphological appearances. IT carcinomas are characterized by cohesive carcinoma cells forming gland-like tubular structures with an expanding or infiltrative growth pattern, like a papillary and well-differentiated adenocarcinoma. However, the cell cohesion is less apparent or absent in DT carcinomas, and cancer cells diffusely spread in the gastric wall as poorly differentiated adenocarcinomas, SRC, or undifferentiated carcinomas [23, 30]. However, there are two types of histological distribution in the MT carcinoma, as described by Lauren [16]. In this study, carcinoma diagnosed as MT made up 21.9% of the whole series in this population, more than that reported by Borch et al. (13%), Lauren (14%), and Stelzner and Emmrich (14.1%), and less than that reported by Carneiro et al. (38.5%) [2, 5, 16, 26]. This discrepancy might be due to the differences in diagnosis criteria, geographical regions, human races, living habits, and so forth. The age and sex distribution of the patients with MT carcinoma was intermediate although IT carcinomas frequently occurred in old men, compared with DT carcinomas.

Morphologically, there are both intestinal and diffuse components in MT carcinomas, and they were found to be significantly larger, more advanced, and more frequently spread into local lymphatic and venous vessels or regional lymph nodes, compared with IT and DT carcinomas in the present study, consistent with other reports [6, 16, 30]. Kozuki et al. [14] found that prominent lymphatic permeation and lymph node metastasis were more frequently observed in MT than in the pure type of gastric carcinomas. It was previously documented that the MT carcinomas showed a deeper infiltration of the gastric wall, a higher metastatic rate to the regional lymph nodes, and the need for higher staging with the TNM system of UICC classification, in contrast to IT and MT carcinomas [26]. These findings suggest that the pathobiological characteristics of the MT carcinomas were more aggressive than the other two types, which accounted for their comparatively poor prognosis observed in the present study although there was no statistical significance. Among the MT carcinomas, five groups were classified according to their growth pattern. Half of the present cases belonged to group I, which means there was diffuse distribution of the intestinal and diffuse component without clear borders in most of the MT cases. In general, groups III and IV of MT carcinomas did not exhibit deep invasion, comparatively high staging or frequent spreading, compared with other types. However, group IV of carcinomas with mucin and signet cells was more advanced with wide spreading, including local vessels and lymph node metastasis. Therefore, surgeons should carefully treat the patients with MT carcinomas in clinical practice because of their aggressive characteristics and poor prognosis, especially those classified as group IV. Furthermore, it was found that the major component in the MT primary foci morphologically paralleled to that in the corresponding lymph node metastasis although the converse situation also appeared. This inconsistent phenomenon could be explained by sampling bias or the metastatic potential of individual carcinoma cells.

Compared with IT carcinomas, the MT counterpart always exhibited more severe characteristics, including invasion into muscularis propria, lymphatic invasion and lymph node metastasis, and high TNM staging in the present study, as observed by Lauren and other studies [16, 26, 30]. To clarify the pheno/genotypes of gastric carcinoma, the expression of Ki-67, caspase-3, p53, FHIT, maspin, VEGF, EMMPRIN, P-GSK3β-ser^9^, MUC-2, MUC-4, MUC-5AC, MUC-6, E-cadherin, and β-catenin was examined by immunostaining. p53, FHIT, and maspin as tumor suppressor genes play important roles in regulating the balance between the proliferation and apoptosis of cancer cells [25, 30, 31, 34, 35]. Ki-67 antigen is present in the nuclei of cells undergoing proliferation and should be regarded as a good marker for cell proliferation [34]. Caspase-3 is responsible for the cleavage of poly (ADP-ribose) polymerase, actin, and sterol regulatory element binding protein and reflects the apoptotic level as a key protease in the cascade reaction of the apoptotic pathway [28]. In vivo and vitro evidences indicated that EMMPRIN and VEGF are involved in angiogenic processes in malignancies [29, 32]. Glycogen synthase kinase-3β (GSK3β) belongs to the serine/threonine protein kinase family and is also involved in regulating the balance between proliferation and apoptosis and can be inactivated via ser-9 phosphorylation by p70 S6 kinase, p90Rsk, Akt, certain isoforms of proteins kinase, and cyclic AMP-dependent protein kinase [1]. MUC-2, MUC-5AC, and MUC-6 are markers for intestinal goblet cells, superficial epithelium, and gastric pyloric gland cells, respectively, which can reflect the mucin secretion and variations in gastrointestinal malignancies. MUC-4 is a heterodimeric glycoprotein complex and expressed in several human epithelial carcinomas [3, 33]. CD44 is a cell surface glycoprotein involved in cell/cell and cell/matrix interactions. CD44 overexpression has been linked to the growth and spread of a range of different types of malignancies [8]. The E-cadherin can interact with β-catenin to form a complex, which is closely linked to cell adhesion and differentiation [19, 27, 36]. In the present study, all of the markers except MUC-5AC showed greater expression in the IT than in the DT carcinomas. Although some evidences indicate that MUC-5AC was strongly expressed in gastric DT carcinoma, in comparison with IT ones, there was no statistical difference in line with our finding [20, 22]. These data suggested that there were significant differences in the proliferation, apoptosis, angiogenesis, mucin production, and cell adhesion between the IT and DT carcinomas and these molecules mechanistically contributed to the molecular distinction in the morphological, behavioral, and histogenic aspects between both types of gastric carcinomas.

In general, DT carcinoma is believed to derive de novo from the peripheral stem cells of gastric gland neck proliferative zone without a recognizable precursor lesion except hereditary diffuse gastric cancer because in situ carcinoma or globoid dysplasia is its precancerous lesion, respectively, according to the Chinese and Western pathologists' opinions [4, 5, 9, 18,30]. When long-standing gastric inflammation causes tissue injury and stem cell failure with time, BMDCs are recruited and engrafted into the tissue stem cell niche, where BMDCs can behave in a way indistinguishable from endogenous tissue stem cells. With continued inflammation and injury, they can undergo sustained proliferation and malignant transformation into IT carcinomas, passing through precancerous stages of metaplasia and dysplasia when genetic defects, such as mutation, deletion, or rearrangement, are accumulated resulting in corruption of the balance between proliferation and apoptosis [17]. Likewise, dedifferentiation of intestinal to diffuse carcinoma had been identified as another histogenic pathway according to the histological heterogeneity of tumor cells, especially in the carcinomas containing moderately and poorly differentiated components with similar morphological appearance and diffuse distribution. If so, it is possible that the distinct components in MT carcinomas arise from the stem cells with common genetic traits and follow different carcinogenic pathways. Conversely, diffuse carcinoma could be derived from heterogeneity of the intestinal counterpart. According to our present data, no differences in the expression of Ki-67, caspase-3, FHIT, CD44, P-GSK3β-ser^9^, MUC-2, MUC-5AC, MUC-6, and membrane β-catenin were observed between the intestinal and diffuse components of MT carcinomas, which supports the possibility of similar origin or dedifferentiation. However, increased expression of EMMPRIN, VEGF, MUC-4, and E-cadherin in the intestinal component compared to the diffuse counterpart also provided evidence that the original carcinoma cells might undergo distinct carcinogenic routes resulting in the morphological distinction of both components. Both the intestinal and diffuse components in MT carcinomas had increased expression of Ki-67, EMMPRIN, and VEGF and reduced E-cadherin. Serum VEGF levels were found to be significantly higher in patients with MT gastric carcinomas than those with pure lesions [10]. It was proposed that MT carcinomas biologically displayed more aggressive behaviors than other types, including decreased cell adhesion, increased proliferation, and angiogenesis.

Most of the evidences in the present data indicated that the pathological behaviors of MT carcinomas were more aggressive than the other types, which was closely linked to the prognosis. It was previously documented that the MT patients' survival is significantly worse than those with IT or DT carcinomas regardless of their location [5]. However, this study demonstrated that there was no significance between the MT and DT carcinoma patients' survival rates although the former was lower than the latter. Additionally, both survival rates were lower than that with IT carcinomas. To avoid sampling bias from a shorter follow-up time, the older cases were chosen for the survival analysis, but they yielded consistent results. Furthermore, Cox's hazard proportional analysis indicated that the invasive depth, venous invasion, lymph node, peritoneal or liver metastasis, and Lauren's classification were independent prognostic factors for gastric carcinomas.

In summary, IT carcinoma, which is positively correlated with favorable prognosis, frequently displayed high levels of proliferation, apoptosis, angiogenesis, mucin production, and cell adhesion. Gastric MT carcinoma showed more aggressive behaviors than IT and DT ones. There was a significant difference in the proliferation, apoptosis, angiogenesis, mucin secretion, and cell adhesion between the IT and DT carcinomas, whereas only a few characteristics were differentially detected in the intestinal and diffuse component of the mixed-type carcinoma, suggesting that different components of MT carcinoma might originate from common stem cells, but follow distinct histogenic pathways. Furthermore, these results confirm that Lauren's classification is significant regarding the histopathogenesis and differentiation and considered as a guide to the clinical treatment of gastric carcinoma.
